# Eating glutinous brown rice twice a day for 8 weeks improves glycemic control in Japanese patients with diabetes mellitus

**DOI:** 10.1038/nutd.2017.26

**Published:** 2017-05-08

**Authors:** T Nakayama, Y Nagai, Y Uehara, Y Nakamura, S Ishii, H Kato, Y Tanaka

**Affiliations:** 1 Division of Metabolism and Endocrinology, Department of Internal Medicine, St Marianna University School of Medicine, Kawasaki, Japan

## Abstract

**Objective:**

We recently reported that eating glutinous brown rice (GBR) for 1 day improved the whole-day glucose profile and postprandial plasma glucose level compared with eating white rice (WR) or standard brown rice. However, it was unknown whether eating GBR could maintain improvement of glycemic control for a longer period. Therefore, we evaluated the effect of GBR intake for 8 weeks on glycemic control in outpatients with diabetes mellitus.

**Methods:**

This was an open-label randomized crossover study in outpatients with type 2 diabetes. Among the 18 subjects registered in this study, 2 were excluded from analysis. After a 1-week observation period while eating WR twice a day, the patients were randomly assigned to two groups. One group ate GBR as a staple food twice a day for 8 weeks and then switched to WR for the next 8 weeks, while the other group ate WR first and then switched to GBR. A mixed meal tolerance test was performed at baseline and after 8 and 16 weeks of dietary intervention to evaluate plasma glucose and serum C-peptide.

**Results:**

None of the subjects failed to complete the study because of disliking the taste of GBR. Hemoglobin A1c (7.5–7.2%, *P*=0.014) and glycoalbumin (20.4–19.4%, *P*=0.029) both decreased significantly when the patients were eating GBR. Additionally, the 30-min postprandial plasma glucose level (194–172 mg dl^−1^, *P*=0.031) and the incremental area under the concentration vs time curve of serum C-peptide (31.3–22.1 ng min ml^−1^, *P*=0.023) during the mixed meal tolerance test were also decreased significantly by intake of GBR. In contrast, there were no changes of glycemic control during the WR period.

**Conclusions:**

We confirmed that GBR was well tolerated for 8 weeks and improved glycemic control in patients with type 2 diabetes.

## Introduction

Asians, especially those from East Asia, are known to have a genetic predisposition to poor insulin secretion by pancreatic β-cells and their insulin secretion is prone to decline with β-cell exhaustion.^1, 2^ Asians have traditionally consumed white rice (WR) as a staple food that provides more than 30% of daily energy intake.^3^ High intake of WR is reported to be associated with an increased risk of type 2 diabetes and metabolic syndrome,^4^ and it has also been reported that this relation is stronger for Asians than Westerners.^5^

In subjects with metabolic syndrome, eating brown rice (BR) led to weight loss, improved endothelial dysfunction and improved glucose and lipid metabolism,^6^ while intake of BR also decreased hemoglobin A1c (HbA1c) in subjects with diabetes mellitus.^7^ When 50 g of WR, corresponding to one-third of the daily intake, is replaced by BR, the risk of developing type 2 diabetes is reduced by 16%.^8^ However, it may be difficult for people to continue eating BR on a daily basis due to its taste and texture, even though BR could prevent or improve diabetes compared with WR.

Rice can be classified as glutinous or non-glutinous. Glutinous rice is stickier and it is mainly used to make rice cakes in Japan, while non-glutinous rice is generally boiled and eaten with the daily meals. The starch in non-glutinous rice (including WR) consists of 20% amylose and 80% amylopectin, whereas the starch in glutinous rice is 100% amylopectin. Since glutinous rice is widely accepted by Japanese people as having a good taste and texture, we expected that patients might prefer glutinous brown rice (GBR) over BR and continue to eat it in daily life. We previously demonstrated that eating GBR for just 1 day improved the whole-day glucose profile compared with eating WR or BR, mainly by reducing postprandial glucose excursion, and GBR also overcame the problem of poor palatability of BR.^9^

Accordingly, the present study was performed to evaluate whether eating GBR twice a day for 8 weeks could improve glycemic control in Japanese patients with type 2 diabetes.

## Materials and methods

### Study design

This randomized, open-label, crossover study was designed to compare the effect on glycemic control of eating two different types of rice as staple food, which were WR (Sato Foods Co., Ltd, Niigata, Japan) and GBR (Nichirei Foods Inc., Tokyo, Japan).

### Subjects

Between August 2015 and June 2016, patients with type 2 diabetes were recruited at the outpatient clinic of St Marianna University Hospital (Kawasaki, Japan). The inclusion criteria were as follows: (1) an age ⩾20 years, (2) stable HbA1c for 6 months (HbA1c>6.0 and<8.9 with ΔHbA1c<0.5%) and (3) treatment with multiple daily insulin injections with or without oral hypoglycemic agents. The exclusion criteria were as follows: (1) an age ⩾75 years, (2) type 1 diabetes, (3) severe renal dysfunction (estimated glomerular filtration rate <30 ml^−1^ min^−1^ per 1.73 m^−2^), (4) women who were pregnant, possibly pregnant, planned to become pregnant, or were breastfeeding and (5) patients who were considered to be ineligible for the study by the attending doctor for other reasons. The treatment of the patients, including oral antidiabetic agents and insulin doses, was not changed throughout the study period.

Written informed consent was obtained from all patients. This study was performed in accordance with the Declaration of Helsinki and was approved by the ethics committee of St Marianna University School of Medicine (No. 2242). This study was also registered with the University Hospital Medical Network Clinical Trials Registry (clinical trial registration number: UMIN000025314).

### Dietary intervention

Among the 18 subjects enrolled in this trial, none were eating BR on a daily basis. In order to maintain a stable intake of rice, a single trained nutritionist interviewed each patient to assess their daily diet. Then the subjects were instructed to eat the specified amount of rice twice a day. After eating WR twice daily for a 1-week observation period, the subjects were randomly assigned to two groups. One group ate GBR twice a day for 8 weeks as a staple food, after which they switched to WR for the next 8 weeks, whereas the other group ate WR first and then GBR.

### Mixed meal tolerance test

At baseline and after 8 and 16 weeks of dietary intervention, a standard meal test (total caloric content of 460 kcal (1.93 MJ), including 53% carbohydrate, 16% protein and 31% fat) was performed to evaluate the plasma glucose and serum C-peptide levels while fasting and at 30, 60, 90, 120 and 180 min postprandially. The test meal was ingested within 15 min. At 0 min of the meal test, the following parameters were also measured: fasting triglycerides, total cholesterol, low-density lipoprotein cholesterol, high-density lipoprotein cholesterol, urea nitrogen, and creatinine, active glucagon-like peptide-1 (GLP-1) and peptide YY (PYY).

### Study end points

The primary end point of this study was the change of HbA1c from baseline after each 8-week dietary intervention period, so HbA1c was measured before and after each period. In order to detect a decrease of HbA1c with a two-sided level of significance of 5% and a power of 80%, a sample size of 16 patients was required. Secondary end points were the changes of glycoalbumin, 1,5-anhydroglucitol, plasma glucose, active GLP-1, PYY and s-CPR. In addition, the total and incremental areas under the glucose or s-CPR vs time curves (AUCs) for 3 h after commencement of the mixed meal tolerance test were calculated by the trapezoidal rule. To investigate whether the oral hypoglycemic agents used by the patients had any influence on the results, a *post hoc* analysis was performed.

### Statistical analysis

The Shapiro–Wilk normality test was used to assess whether variables had a normal distribution. Categorical variables were expressed as numbers or percentages, while continuous variables were expressed as the mean±s.d. or s.e.m. One-way analysis of variance was used to confirm a crossover order effect. The paired *t*-test was employed to assess changes of variables from baseline. All analyses were performed with JMP version 12 (SAS Institute Inc., Cary, NC, USA). Differences were considered to be significant if the probability value (*P*) was less than 5%.

## Results

**Figure 1:**
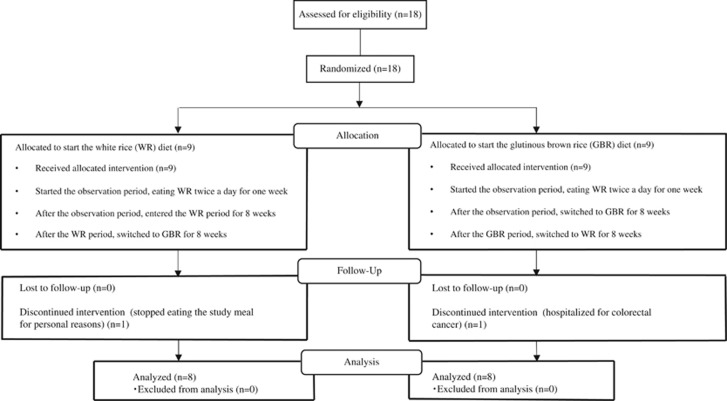
Flow diagram of subject disposition.

Among the 18 subjects registered in this study, 2 were excluded from analysis because of personal problems (*n*=1) and emergency hospitalization for colorectal cancer (*n*=1). The other 16 subjects (12 men and 4 women) completed the study and formed the per protocol set for analyses (Figure 1). None of the subjects dropped out of the study because of disliking the taste of GBR.

Baseline characteristics of the 16 subjects are shown in Table 1. Their average age was 64.0±8.8 (45–74) years and average body mass index was 25.7±5.6 (14.9–36.2) kg m^−2^. The mean duration of diabetes was 14.7±10.3 (2–33) years and the mean dose of insulin was 34.4±16.0 (12–60) units per day. The average energy intake per meal from GBR and WR was 277±75 kcal (1157±312 kJ).

**Figure 2:**
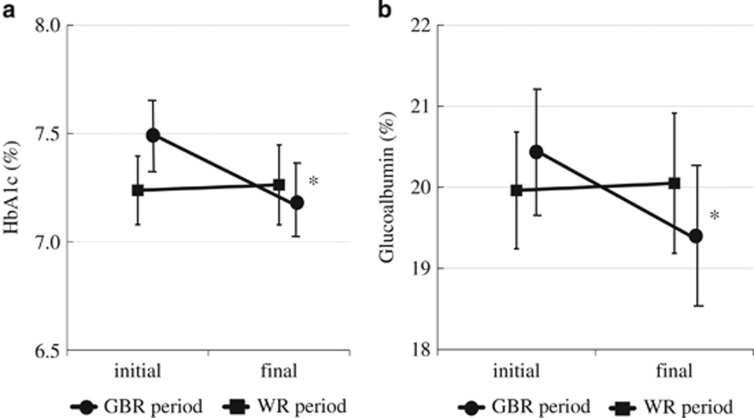
Changes of HbA1c (**a**) and glycoalbumin (**b**) during the GBR period (circles) and the WR period (squares). Data are expressed as the mean±s.e. *P<0.05 vs baseline.

**Figure 3:**
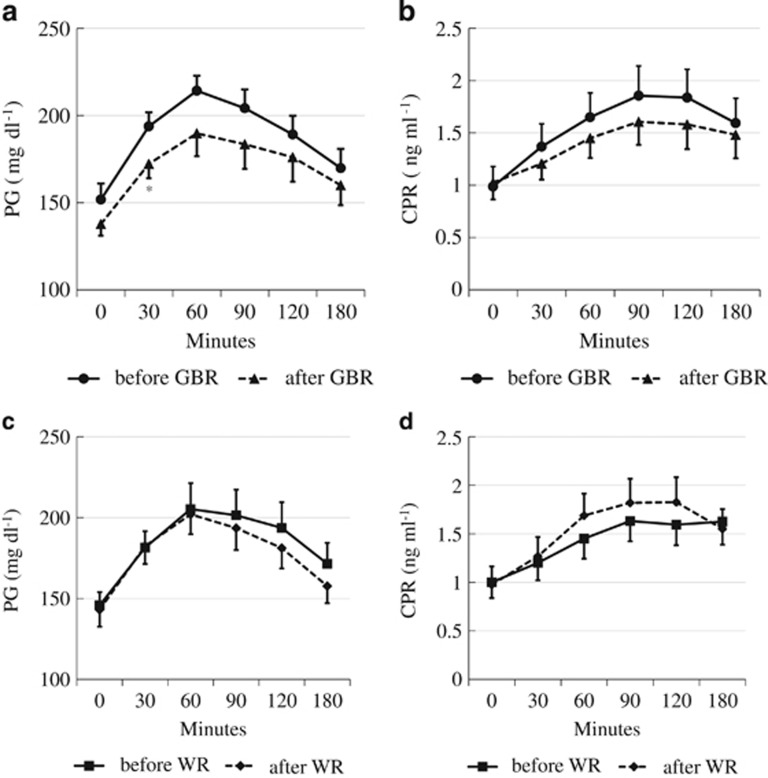
Profile of plasma glucose (**a**,**c**) and C-peptide (**b**,**d**) during the mixed meal tolerance test at baseline (black circles) and after 8 weeks (black triangles) of GBR intake (**a**, **b**) or WR intake (**c**,**d**). Data are expressed as the mean±s.e. *P<0.05 vs baseline.

In the group eating GBR first, the baseline HbA1c was 7.5±0.5%. After 8 weeks of eating GBR twice daily, HbA1c showed a decrease to 7.1±0.5%. After switching to WR for 8 weeks, the HbA1c of this group remained at 7.1±0.5%. In the group that ate WR first, HbA1c was 7.4±0.7% at baseline, 7.5±0.9% after 8 weeks of eating WR twice daily and 7.3±0.8% after switching to GBR for the subsequent 8 weeks. A crossover order effect was not detected by one-way analysis of variance (*P*=0.409). When pooled data for GBR and WR were compared, there was a significant decrease of HbA1c from 7.5±0.2 to 7.2±0.2% (*P*=0.014) when the subjects were eating GBR (Figure 2a), while there was no significant change while they were eating WR (7.2±0.2–7.3±0.2%, *P*=0.848; Figure 2b). Dietary intake of GBR also resulted in a significant decrease of glycoalbumin from 20.4±0.8 to 19.4±0.9% (*P*=0.029), while there was no change with WR (from 20.0±0.7 to 20.1±0.9%, *P*=0.875). The profiles of plasma glucose and serum C-peptide during the mixed meal tolerance test are displayed in Figure 3. In comparison with baseline, plasma glucose levels were lower when the subjects were eating GBR, and there was a significant reduction from 193.8±8.1 mg dl^−1^ (10.7±0.4 mmol l^−1^) to 172.3±8.2 mg dl^−1^ (9.5±0.4 mmol l^−1^; *P*=0.031) at 30 min. Although the serum C-peptide level did not differ significantly from baseline at each time point of assessment, the incremental AUC_C-peptide_ was significantly smaller when the subjects were eating GBR from 31.3±5.6 to 22.1±4.5 ng min ml^−1^ (*P*=0.023). On the other hand, there were no significant changes of plasma glucose and serum C-peptide (including the total and incremental AUC) while the subjects were eating WR. Furthermore, there was no significant change of body mass index throughout the entire 16-week study period. Other parameters also showed no significant changes during the study period, including plasma levels of T-C, high-density lipoprotein, triglycerides and BUN, and the estimated glomerular filtration rate. No adverse events occurred in any of the 16 subjects who completed the study.

**Figure 4:**
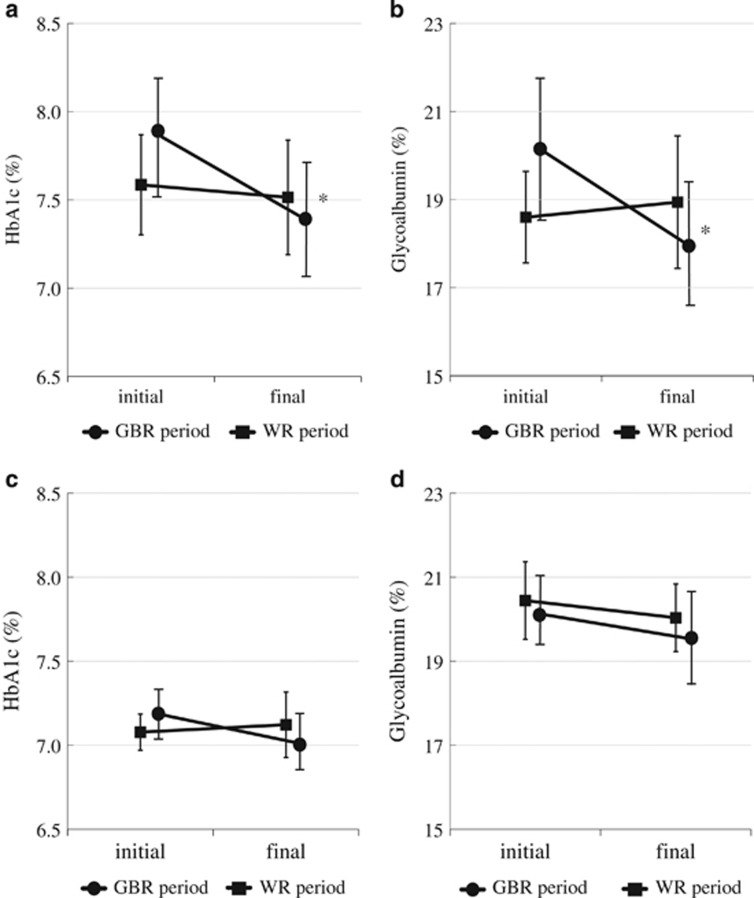
Changes of HbA1c (**a**,**c**) and glycoalbumin (**b**,**d**) during the GBR period (circles) and the WR period (squares) in patients with (**a**,**b**) or without (**c**,**d**) concomitant use of DPP-4 inhibitors. Data are expressed as the mean±s.e. *P<0.05 vs baseline.

*Post hoc* analysis showed a significant decrease of HbA1c (from 7.9±0.8 to 7.4±0.8%, *P*=0.013) and glycoalbumin (from 20.2±4.3 to 17.9±3.8%, *P*=0.002) when eating GBR in patients using DPP-4 inhibitors (*n*=7; Figures 4a and b), while there were no significant decreases when eating GBR without DPP-4 inhibitor therapy (*n*=9; Figures 4c and d). In contrast, there was no difference in the effect of WR on glycemic control between the subgroups with or without DPP-4 inhibitors (data not shown). Plasma levels of active GLP-1 and PYY also increased in patients using DPP-4 inhibitors when eating GBR (Supplementary Figure). With respect to insulin sensitizers, there were no significant differences in the effects of GBR or WR between patients with (*n*=8) and without (*n*=8) metformin (data not shown).

## Discussion

We previously reported that eating GBR for even 1 day reduced postprandial glucose excursion compared with intake of WR. In the present study, postprandial plasma glucose, glycoalbumin and HbA1c were significantly decreased (by 21.5 mg dl^−1^, 0.3% and 1.1%, respectively) after 8-week ingestion of GBR twice a day, demonstrating that the single-day improvement of the plasma glucose profile could be maintained for at least 8 weeks. The incremental AUC_C-peptide_ was also significantly smaller while the subjects were eating GBR, despite no change of weight. These results suggested that improvement of glycemic control by intake of GBR may not have been due to increased insulin secretion. However, the mechanisms involved are presently unknown.

It was interesting that this study demonstrated a significant difference in the effect of GBR between patients with and without concomitant use of DPP-4 inhibitors, which inhibit the breakdown of GLP-1. Why was there greater improvement of glycemic control by GBR in patients using DPP-4 inhibitors? A possible explanation is that GBR might stimulate GLP-1 secretion secondary to an increase of short-chain fatty acids produced from dietary fiber by the gut microbial flora. A previous study showed that eating whole grains for 6 weeks improved gut flora and increased short-chain fatty acid levels.^10^ It was also reported that the short-chain fatty acid propionate stimulates secretion of GLP-1 and PYY via free fatty acid receptor 2 in rodents.^11^ GBR has a higher content of dietary fiber compared with WR (Supplementary Table). When we compared plasma levels of active GLP-1 and PYY before and after each period, both were increased by intake of GBR (Supplementary Figure), although the changes were not significant due to the small sample size. Therefore, DPP-4 inhibitor therapy could have an additive effect on the benefits of GBR.

GBR consists of bran, embryo Buda germ and endosperm. Bran also contains some substances, such as magnesium and γ-oryzanol, that may improve glycemic control.^12, 13^ Insufficient dietary intake of magnesium may contribute to exacerbation of insulin resistance due to inhibition of the autophosphorylation of insulin receptors.^14, 15^ One pack of GBR (113 g) contains 72.5 mg of magnesium, while the same amount of WR only contains 2.3 mg. Bran is also rich in γ-oryzanol.^12^ It has been reported that a single oral dose of γ-oryzanol improves glycometabolic parameters and insulin resistance in non-insulin-dependent obese patients with type 2 diabetes.^12, 16^ We confirmed that one pack of GBR contains 31 mg of γ-oryzanol, although endosperm does not contain γ-oryzanol. These features of GBR may partly explain the improvement of glycemic control by intake of this type of rice in the present study.

Eating GBR overcomes the problem of poor palatability associated with BR. We previously reported that the palatability of GBR was similar to that of WR, based on the results of a questionnaire survey after ingestion of the different types of rice for 1 day each.^9^ In that study, the subjects reported striking differences of taste, texture and consumability between BR and GBR in spite of the similar appearance of these two types of rice. In the present study, there were no dropouts because of unacceptability of the taste of GBR. This is the first study to demonstrate that intake of GBR is tolerable and maintains efficacy for at least 8 weeks.

The present study had several limitations including a small sample size. Although a double-blind, randomized study would have been desirable, we used an open-label, crossover design because of the difficulty of blinding the different types of rice. Accordingly, we cannot exclude the possible influence of bias on our results. In addition, given the limitations of *post hoc* analysis, a randomized prospective study is needed to confirm the better response to GBR of patients treated with DPP-4 inhibitors. Furthermore, we did not assess insulin sensitivity, although a change of insulin sensitivity might have been associated with the improvement of glycemic control by GBR. Since we detected a decrease of the incremental AUC_C-peptide_ during the GBR period of the present study that suggested improvement of insulin sensitivity, this warrants further evaluation. Despite these limitations, the present findings could be useful for assisting patients with diabetes to make decisions about their diet.

In conclusion, eating GBR twice a day for 8 weeks was well tolerated with respect to palatability and led to sustained improvement of glycemic control in patients with type 2 diabetes.

## Figures and Tables

**Table 1:** Characteristics of the subjects (*n*=16)

n (male/female)	(n)	12/4
Age	(years)	64±8.8
Body mass index	(kg m^−2^)	25.7±5.6
HbA1c	(%)	7.5±0.6
Glycoalbumin	(%)	20.7±2.6
1,5-anhydroglucitol	(μg ml^−1^)	6.9±3.1
Blood urea nitrogen	(mg dl^−1^)	14.9±3.7
Creatinine	(mg dl^−1^)	0.8±0.2
eGFR	(ml ^−1 ^min^−1^ per 1.73 m^2^)	76.6±19.5
Total cholesterol	(mg dl^−1^)	175.1±37.2
LDL cholesterol	(mg dl^−1^)	101.8±28.9
HDL cholesterol	(mg dl^−1^)	53.8±18.6
Triglycerides	(mg dl^−1^)	101.1±50.5
Fasting blood glucose	(mg dl^−1^)	154.1±22.9
Fasting serum C-peptide	(ng ml^−1^)	1.0±0.8
		
*Diabetes treatment*		
Total insulin dose	(units per day)	34.4±16.0
MDI only	(% (*n*))	37.5 (6)
MDI + oral hypoglycemic agents	(% (*n*))	62.5 (10)
Biguanide		50 (8)
Sulfonylurea		0 (0)
Glinide		0 (0)
α-glucosidase inhibitor		18.8 (3)
Thiazolidinedione		0 (0)
DPP-4 inhibitor		43.8 (7)
SGLT2 inhibitor		0 (0)

Abbreviations: eGFR, estimated glomerular filtration rate; HbA1c, hemoglobin A1c; HDL, high-density lipoprotein; LDL, low-density lipoprotein; MDI, multiple daily injection. Data are the mean±s.d. or % (*N*).
