# Supplementary figures and images for: Eating glutinous brown rice twice a day for 8 weeks improves glycemic control in Japanese patients with diabetes mellitus
Source: Nutr Diabetes. 2017 May 8;7(5):e273–. doi: 10.1038/nutd.2017.26 (PMC5518808; doi:10.1038/nutd.2017.26)

## Slide 1
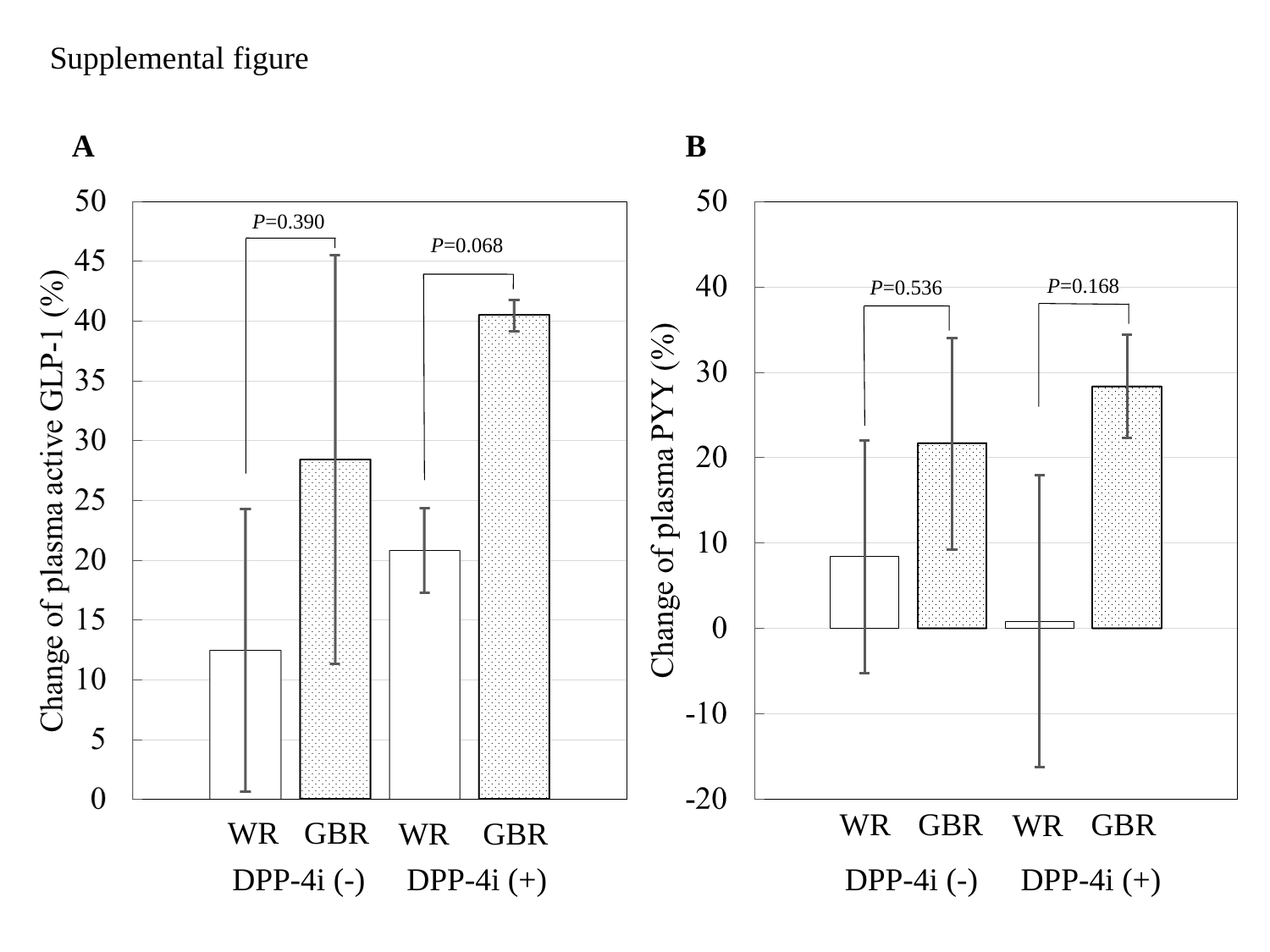

Supplemental figure
A
B
P=0.390
P=0.068
P=0.168
P=0.536
WR
GBR
GBR
WR
WR
GBR
WR
GBR
DPP-4i (-)
DPP-4i (+)
DPP-4i (-)
DPP-4i (+)

Supplement: Supplementary Figure [file nutd201726x1.ppt]
